# Pseudouridine synthase 1 deficient mice, a model for Mitochondrial Myopathy with Sideroblastic Anemia, exhibit muscle morphology and physiology alterations

**DOI:** 10.1038/srep26202

**Published:** 2016-05-20

**Authors:** Joshua E. Mangum, Justin P. Hardee, Dennis K. Fix, Melissa J. Puppa, Johnathon Elkes, Diego Altomare, Yelena Bykhovskaya, Dean R. Campagna, Paul J. Schmidt, Anoop K. Sendamarai, Hart G. W. Lidov, Shayne C. Barlow, Nathan Fischel-Ghodsian, Mark D. Fleming, James A. Carson, Jeffrey R. Patton

**Affiliations:** 1 Integrative Muscle Biology Laboratory, Department of Exercise Science, Public Health Research Center, University of South Carolina, Columbia, SC 29208, USA; 2 Department of Pathology, Microbiology, and Immunology, University of South Carolina, School of Medicine, Columbia, SC 29209, USA; 3 Department of Drug Discovery and Biomedical Sciences, South Carolina College of Pharmacy, University of South Carolina, Columbia, SC 29208, USA; 4 Medical Genetics Institute, Ahmanson Department of Pediatrics, Steven Spielberg Pediatric Research Center, Cedars-Sinai Medical Center, David Geffen School of Medicine at UCLA, Los Angeles, CA 90048, USA; 5 Department of Pathology, Boston Children’s Hospital, Boston, MA 02115, USA; 6 Animal Resource Facility, University of South Carolina, Columbia, SC 29208, USA

## Abstract

Mitochondrial myopathy with lactic acidosis and sideroblastic anemia (MLASA) is an oxidative phosphorylation disorder, with primary clinical manifestations of myopathic exercise intolerance and a macrocytic sideroblastic anemia. One cause of MLASA is recessive mutations in *PUS1*, which encodes pseudouridine (Ψ) synthase 1 (Pus1p). Here we describe a mouse model of MLASA due to mutations in *PUS1*. As expected, certain Ψ modifications were missing in cytoplasmic and mitochondrial tRNAs from *Pus1*^−/−^ animals. *Pus1*^−/−^ mice were born at the expected Mendelian frequency and were non-dysmorphic. At 14 weeks the mutants displayed reduced exercise capacity. Examination of tibialis anterior (TA) muscle morphology and histochemistry demonstrated an increase in the cross sectional area and proportion of myosin heavy chain (MHC) IIB and low succinate dehydrogenase (SDH) expressing myofibers, without a change in the size of MHC IIA positive or high SDH myofibers. Cytochrome *c* oxidase activity was significantly reduced in extracts from red gastrocnemius muscle from *Pus1*^−/−^ mice. Transmission electron microscopy on red gastrocnemius muscle demonstrated that *Pus1*^−/−^ mice also had lower intermyofibrillar mitochondrial density and smaller mitochondria. Collectively, these results suggest that alterations in muscle metabolism related to mitochondrial content and oxidative capacity may account for the reduced exercise capacity in *Pus1*^−/−^ mice.

Pseudouridine (Ψ), an abundant base modification^1^ found in snRNAs^2^, tRNAs^3^, and rRNAs^4^, strengthens base-pairing^5^,^6^,^7^,^8^ and stabilizes base stacking^9^. Ψ in snRNAs is required for pre-mRNA splicing^10^,^11^,^12^,^13^ and Ψ in tRNAs is involved in stop codon suppression^14^, recoding efficiency^15^, and translational specificity^16^. Furthermore, in the large ribosomal subunit rRNA, many of the Ψs are clustered in the peptidyl-transferase center and other functional regions^17^.

Pseudouridine synthases are responsible for the formation of Ψ in RNA in either a site-specific or snoRNA guide RNA-dependent manner. Pseudouridine synthase 1 (Pus1p) is a member of the TruA family^18^,^19^, and members of this family, and all other pseudouridine synthases, have a conserved aspartate in the active site^20^,^21^,^22^,^23^,^24^,^25^,^26^. Of the site-specific synthases, Pus1p is one of the best studied and, at a minimum, is responsible for the pseudouridylation of tRNAs^18^,^27^,^28^,^29^, U2 snRNA^28^,^30^, and steroid receptor RNA activator RNA (SRA)^26^,^31^,^32^,^33^. Pus1p is a co-activator of nuclear receptor-dependent transactivation, modifying SRA as a part of a promoter-bound complex^26^. The capacity of Pus1p to modify several different types of RNA is unusual, compared with other pseudouridine synthases, and may be the result of its lack of strict sequence recognition requirements^34^. Instead it relies on local secondary structure and the appropriate presentation of the uridine to be modified in the active site^33^,^34^,^35^.

Mitochondrial myopathy with lactic acidosis and sideroblastic anemia (MLASA; OMIM #600462 and 613561) is an autosomal recessive oxidative phosphorylation disorder that affects muscle and bone marrow, resulting primarily in exercise intolerance and anemia^36^,^37^,^38^,^39^. MLASA may also be associated with mental retardation and/or craniofacial abnormalities^37^,^39^. MLASA is the result of mutations in *PUS1*^40^,^41^ or *YARS2*, which encodes mitochondrial tyrosyl-tRNA synthetase^42^,^43^,^44^,^45^. Cell lines derived from patients with MLASA who have mutations in *PUS1*, lack Pus1p activity as well as Pus1p-dependent pseudouridinylation^46^ and have alterations in gene expression^47^.

Although Ψ is important in the structure and function of RNA, it is unclear why the loss of Pus1p activity would result in the set of symptoms that are hallmarks of MLASA. Deletion of *PUS1* in yeast results in no obvious phenotype^27^. However, deletion of *PUS1* in combination with loss of *PUS4*, which modifies position 55 in nearly all tRNAs in yeast and is itself non-essential, is lethal, possibly due to a defect in nuclear export of certain tRNAs^48^. The loss of Pus1p in *C. elegans* results in a slight delay in maturation that does not result in a noticeable change in any other aspect of nematode morphology, locomotion, or metabolism^49^. In this report, we describe the phenotype of mice with a germline deletion of *PUS1.*

## Materials and Methods

### Assurances

This study was carried out in strict accordance with the recommendations in the Guide for the Care and Use of Laboratory Animals of the National Institutes of Health. The protocol (1775-100563-013012) was approved by the Institutional Animal Care and Use Committee (IACUC) of the University of South Carolina (Assurance number A3049-01) and that the methods were carried out in accordance with the approved protocol. At Children’s Hospital the protocol number 13-08-2476R was approved by the IACUC at Children’s Hospital Boston (Assurance number is A3303-01) and that the methods were carried out in accordance with the approved protocol. Human subjects were not used in these studies, at either the University of South Carolina or Children’s Hospital Boston.

### *PUS1* gene targeting

**Figure 1:**
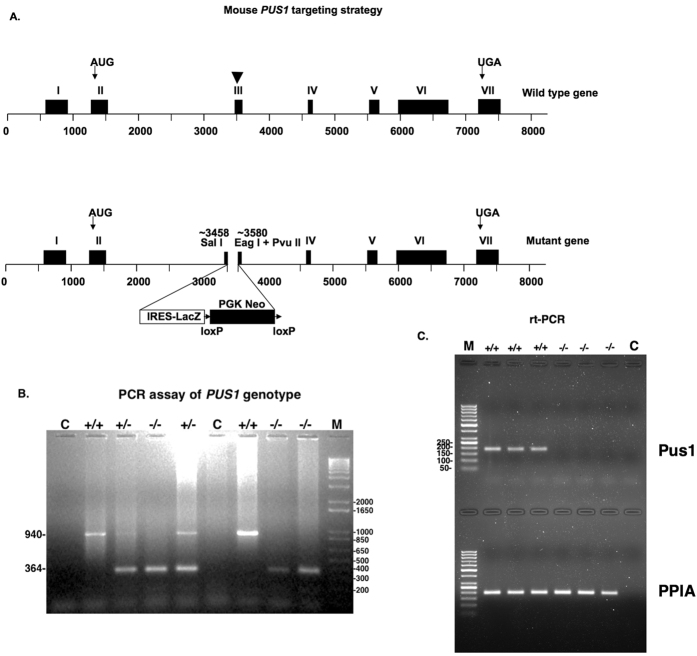
Mouse *Pus1* gene targeting strategy and assay of genotype. In panel (**A**) the targeting strategy is presented with a diagram of the wild-type gene on the top, indicating the positions of the initiator codon in exon II and the stop codon in exon VII with arrows. Exon III is indicated with an arrowhead because this exon contains the codon for the Asp that is essential for activity (see Results). This exon was targeted for disruption and the resulting mutant gene is shown in the lower diagram. In panel (**B**) an example of an ethidium bromide-stained gel employed to determine the genotypes of the mice is shown. The lane labeled C denotes a control where no DNA was added to the PCR reaction, **+/+** is the result when DNA from wild-type mice is used, **+/−** is the result when DNA from heterozygous mice is used, and −/− is the result when DNA from homozygous mutant mice is used. M denotes the 1Kb+ marker (Promega, Madison, Wisconsin) with sizes in bp on the right. The sizes the bands resulting from the wild-type (940 bp) and mutant (364 bp) genes are indicated on the left of the panel. In panel (**C**) a 1.5% agarose gel stained with ethidium bromide showing the results of real time-PCR reactions of mRNA from three *Pus1*^+/+^ and three *Pus1*^−/−^ mice with primers specific for Pus1p mRNA in the top portion of the gel and primers specific for PPIA in the bottom portion. The marker in the far-left lanes is the GeneRuler 50 bp DNA ladder (Life Technologies) with sizes represented on the left in the top portion. The far right lanes in both portions of the gel are the results of using no cDNA in the PCR reaction.

The targeting vector (Fig. 1) was constructed using standard recombinant DNA protocols. Briefly, the 5′ arm (2486 bp) was generated by PCR^50^ using primers: 5′TTTGTCGACATTTAAATAGAACCCAAGACTGTCCTG and 5′CTAGTCGACGACCCCAGATTCCTCTAAAG. The cloned fragment was inserted into the SalI site of pNTRLacZPGKNeoLoxp. The 3′ arm (2379 bp) was prepared similarly using the primers 5′TTTCGGCCGACAGCTGAAAGGTATGTGTCCTACTTG and 5′TTTGCGGCCGCAGTTTAAACCTGACACTGTGGTTCTGGTG and an EagI fragment was cloned into the NotI site in the vector. A thymidine kinase (TK) cassette was incorporated into the vector and the resulting targeting construct was linearized with Swa I and electroporated into mouse AB2.2 (129 SV/EV) ES cells (Darwin Transgenic Core, Baylor College of Medicine https://www.bcm.edu/research/advanced-technology-core-labs/lab-listing/mouse-embryonic-stem-cell-core/). Resulting G418 resistant/TK minus cell lines were screened for recombination^51^. Correctly targeted ES cell lines were identified, expanded, and two were injected into C57BL/6J blastocysts to generate chimeric mice and germline transmission obtained by breeding to C57BL/6 mice. The neomycin resistance cassette was removed by breeding to CMV-CRE mice (Jackson Laboratory- B6.C-Tg(CMV-cre)1Cgn/J, stock# 006054) and confirmed using the following primers: Pus1ex3F 5′TTGCAGAAGTGATGGTCAGC, Pus1ex3 R 5′AGTAGAAGCCGCAGGCAAGT, and Pus1-Neo KO F2 5′CCCCCTGAACCTGAAACATA. The wild-type allele produces a 249 bp fragment and mutant allele without the Neo cassette produces a 622 bp fragment. Genotypes were determined using the following primers: mPus1PCRscreenFor1, 5′TTTTGGGGAGTGGTTCTGAC; mPus1PCRscreenRev1, 5′GAAAGCAAAGGCCAGTGAAG; LacZFor, 5′TTCACTGGCCGTCGTTTTACAACGTCGTGA; and LacZRev, 5′ATGTGAGCGAGTAACAACCCGTCGGATTCT. The amplicons from the wild-type *PUS1* locus and the LacZ in the mutant locus result in 940 bp and 364 bp fragments, respectively. The allele was subsequently backcrossed onto C57BL/6J by heterozygous x wild-type matings for greater than 5 generations prior to experimentation.

### Assay of tRNA pseudouridylation

The CMCT/primer extension method^52^ was employed to assay for the presence of Ψ in a specific tRNA in total RNA from mouse kidney. The following primers we used: muscytoIleAAURev2Fluor 5′IRDye^®^700GATCGAACCCGCGACCTTGG and musmtHisGUGRevFluor 5′IRDye^®^800GGTTTATTTCCTGTTGTCAG. Amplicons were sequenced with the fmole^®^ sequencing kit (Promega) according to the manufacturer’s recommendations. The template for the tRNA^Ile^(AAU) sequencing reactions was a plasmid containing the entire coding sequence of the mouse cytoplasmic tRNA^Ile^(AAU) gene in pGEMT. The template for the mouse mitochondrial tRNA^His^(GUG) sequencing reactions was a gel purified PCR fragment, 65 bp in length, amplified from mouse genomic DNA with the following primers: musmtHisGUGFor, 5′GTGAATATAGTTTACAAAAAAC and musmtHisGUGRev1, 5′GAATAAGGAGGTTTATTTCC, using standard PCR protocols^50^. The samples were electrophoresed on 15% acrylamide:bis acrylamide (19:1) denaturing (8.3 M urea) gels and the fluorescence detected on a Li-Cor Odyssey CLX infrared imager (Instrumentation Resource Facility at the University of South Carolina School of Medicine (http://irf.med.sc.edu)) with no further treatment of the gel^53^. 1-cyclohexyl-3(2-morpholinoethyl) carbodiimide metho-*p*-toluenesulphonate (CMCT; was purchased from Sigma-Aldrich. The fluorescently-labeled primers were purchased from Integrated DNA Technologies (IDT; Coralville, Iowa) with IRDye^®^700 or IRDye^®^800 at the 5′ end, and these primers were HPLC purified by IDT.

### Real time PCR

Total RNA was isolated from muscle tissue and cDNA was generated using iScript mix and Reverse transcriptase (BioRad, Inc.) as described in package inserts using 1 μg of total RNA per reaction, 1X iScript reaction mix and a 1:20 dilution of the iScript reverse transcriptase. The reactions were incubated at 25 °C for 5 minutes, at 42 °C for 30 minutes, and at 85 °C for 5 minutes. The cDNA was diluted 1:100 and 6 μl were used per real-time PCR reaction with iTaq Universal SYBR Green Supermix (BioRad) diluted 1:1 and 500 nM of each primer^54^. The two primers used to amplify from *PUS1* mRNA are: forward, 5′AGCATCCTGCAAAGAGGGTC and Reverse, 5′GCCTGGACTAAGGCCGATAC. For peptidylprolyl isomerase A (PPIA) mRNA the two primers used were: forward, 5′GCAAATGCTGGACCAAACAC and reverse, 5′AGAGCTGTCCACAGTCGGAA. The parameters for the *PUS1* primers were 95 °C for 3 mins, then 95 °C for 30 sec, 60.5 °C for 30 secs, 40×. The parameters for the PPIA reactions were the same except the annealing/extension temperature was increased to 63.9 °C.

### DNA microarrays and gene expression analyses

Total RNA for gene expression analysis was isolated from frozen tissues (brain (cross section through whole brain), heart (cross section through atrium and ventricle), kidney (cross section), liver, and skeletal muscle, gastrocnemius, red (slow) and white (fast), using the mirVana^TM^ PARIS^TM^ kit. RNA quality was assessed using an Agilent 2100 Bioanalyzer and RNA Integrity Numbers (RIN) ranged from 7.1 to 9.0.

Microarray experiments were performed using Agilent’s platform. Total RNA samples were amplified and labeled using Agilent’s Low Input Quick Amp Labeling Kit according to the manufacturer recommendations. Briefly, samples contained 200 ng (brain, kidney, liver and white (fast) skeletal muscle) or 100 ng (heart and red (slow) skeletal muscle) of total RNA were converted into cDNA using a poly-dT primer that also contains the T7 RNA polymerase promoter sequence. Subsequently, T7 RNA polymerase was added to cDNA samples to amplify the original mRNA molecules and to simultaneously incorporate cyanine 3- or cyanine 5-labeled CTP (cRNA) into the amplification products. In addition, Agilent RNA spike-in controls (Cat. # 5188-5279) were added to samples prior to cDNA synthesis and used as experimental quality controls. In the next step, labeled RNA molecules were purified using Qiagen’s RNeasy Mini Kit. After spectrophotometric assessment of dye incorporation and cRNA yield, samples were stored at −80 °C until hybridization. Labeled cRNA samples were hybridized to SurePrint G3 Mouse GE 8 × 60 K Microarrays (Cat. # G4852A-028005) at 65 °C for 17 hours using Agilent’s Gene Expression Hybridization Kit according to the manufacturer’s recommendations. Wild-type and *Pus1*^−/−^ (4 of each per tissue) samples were hybridized in a dye swap design. After washes, arrays were scanned using an Agilent DNA Microarray Scanner System.

Data was extracted from images with Feature Extractor Software version 10.7.3.1 (Agilent). In this process, background correction using additive and multiplicative detrending algorithms was performed. In addition, linear and LOWESS methods were used for dye normalization. Subsequently, data was uploaded into GeneSpring GX (Agilent), was log2 transformed, quantile normalized, and base line transformed using the median of all samples. Then, data was filtered by flags in a way that 75% of the samples in at least one of the two treatment groups have a “detected” flag and gene set enrichment/leading edge analysis was performed with the GSEA software^55^,^56^. The microarray data has been entered in the GEO database with the Superseries record number GSE77823.

### Western blots

Mouse tissues (brain, heart, liver, red and white gastrocnemius skeletal muscle) in RIPA buffer (50 mM Tris pH 8, 150 mM NaCl, 0.1% SDS, 1.0% NP-40, 0.5% sodium deoxycholate, and 1:200 dilution of protease inhibitor (Sigma P8340)) were homogenized in Dounce homogenizers and extracted at 4 °C for 90 minutes on an orbital shaker. After a 12,000 rpm spin for 20 minutes at 4 °C (10,000xg)^57^, the protein concentration in the supernatant was determined and 40 μg of each sample was loaded on either a 12% polyacrylamide (29:1, acryl:bis) gel or an Any kD gel (BioRad, Inc). The gels were transferred to PVDF membrane, blocked, and reacted with primary and secondary antibodies as appropriate for the particular experiment.

### Transmission electron microscopy

Liver and muscle samples were fixed at 4 °C in 2.5% glutaraldehyde in phosphate buffered saline and then processed using conventional techniques^58^,^59^. The samples were imaged on a JEOL 200CX transmission electron microscope in the Instrumentation Resource Facility (see above). Intermyofibrillar (IMF) density and size were determined by tracing the outline of IMF mitochondria at 10,000× magnification using Image J software (NIH, Bethesda, MD, USA). IMF mitochondria were defined as mitochondria greater than 1 μm from the plasma membrane and clearly integrated within the myofilaments^60^. IMF mitochondrial density is expressed as the percent of total myofiber area. An average of 105 mitochondria were traced per animal (N = 3/group) in a blinded fashion.

### Run to Fatigue

The run to fatigue test was performed to examine endurance exercise capacity^61^. Mice were acclimated to the treadmill (both male and female experimenters were present for all tests) and the test began at 5 m/min at 5% grade. The speed was then increased after 5 minutes to 10 m/min, followed by 15 m/min after another 5 minutes. Thirty minutes into the test the speed was adjusted to a maximum of 25 m/min. The maximum time, in seconds, that the mice could run with gentle prods during the entire trial was recorded as the run to fatigue time.

### Rotorod Testing

Neuromuscular coordination was assessed with the Rotorod (Columbus Instruments, Columbus, OH) as previously described^61^. Mice were placed on the Rotorod apparatus and subjected to a ramping protocol test. This protocol increases speed from 0 to 25 rpm over a period of 90 seconds and continued at 25 rpm until the maximum possible time for each trial, 300 seconds, was reached. The average and maximum speed (rpm) and time (seconds) for three trials were recorded. Each mouse performed three trials with 2 minutes rest between trials.

### Grip Strength

Total grip strength of the forelimbs was measured with the Grip Tester (Columbus Instruments, Columbus, OH) as previously described^61^,^62^. Mice were placed on a 45° angled grid connected to a force transducer. Mice were then pulled away from the grid by the tail until they could no longer hold the grid. Each mouse performed 2 sets of 5 trials with two minutes rest between sets. The highest and lowest values were removed, and the average values in Newtons (N) were calculated.

### Tissue collection

Mice were euthanized by CO_2_ asphyxiation. Hindlimb skeletal muscles and organs were excised, rinsed in PBS, snap-frozen in liquid nitrogen, and stored at −80 °C until further analysis. The right tibialis anterior (TA) was placed in optimal cutting temperature (OCT) solution and frozen in isopentane cooled in liquid nitrogen for histological analysis.

### Tibialis anterior morphology

Serial transverse muscle sections (10 μm) were cut from the mid-belly of the tibialis anterior on a cryostat at −20 °C and stored at −80 °C until further analysis. Hematoxylin and eosin (H&E) staining was performed on cross-sections to examine muscle morphology. Digital photographs were taken from each cross-section at 40x magnification with a Nikon spot camera. For cross-sectional area, approximately 125 fibers per animal were traced with imaging software (ImageJ-NIH) in a blinded fashion. Centralized nuclei, defined as nuclei found equidistant from a well-defined sarcolemma, were quantified from these images, and is expressed as the percent of centralized nuclei per total muscle fibers. The extracellular matrix area was quantified as previously described^62^,^63^. Images containing well-defined sarcolemma were traced and the extracellular matrix is expressed as the percentage of whole muscle.

### Immunohistochemistry for myosin heavy chain IIA and IIB

Immunohistochemistry for myosin heavy chain IIA and IIB was performed as previously described^64^. Transverse muscle sections were air dried for 10 minutes, fixed in cold acetone for 1 minute, and washed in PBS for 5 minutes. Cross-sections were quenched in 0.3% H_2_O_2_-methanol solution for 20 minutes and rinsed in PBS for 5 minutes, three times. Sections were then blocked in 10% normal goat serum (Vectastain ABC kit, Vector Laboratories, Burlingame, CA) in PBS for 1 hour at room temperature and then incubated overnight at 4 °C with primary antibodies (IIA: SC-71 and IIB: BF-F3). Following overnight incubation, the sections were then washed three times in PBS. Secondary antibodies were applied to the sections and incubated at 37 °C for 1 hour. The sections were washed three times and the avidin-biotin complex system (ABC: Vector Laboratories) was used to detect the biotinylated secondary antibody (30 minutes at RT). Sections were washed three times in PBS and visualized by incubating in DAB solution for 6 minutes (Vectastain DAB kit). Slides were washed in dH_2_O, dried and mounted with Permount. Digital images were acquired as previously described (see *Tibialis Anterior Morphology* above).

### Succinate Dehydrogenase Staining

Succinate dehydrogenase (SDH) enzyme activity was performed as previously described to determine myofiber oxidative capacity^64^. Frozen sections were air dried at room temperature for 10 min, followed by incubation in a solution containing 0.2 M phosphate buffer (pH 7.4), 0.1 M MgCl_2_, 0.2 M succinic acid, and 2.4 mM nitroblue tetrazolium at 37 °C for 45 min. Sections were then washed in dH_2_O for 3 min, dehydrated in 50% ethanol for 2 min, and mounted for viewing with mounting media. Digital images were acquired as previously described (see *Tibialis Anterior Morphology* above). The percentage of SDH positive fibers was determined at 20×. The SDH staining intensity was determined by subtracting the background from each slide to create an integrated optical density for each myofiber. The percentages of each stain (dark and light) were quantified and expressed as percent of total muscle fibers. High and low SDH activity myofibers were traced at a 40× magnification in a blinded fashion. Approximately 100 myofibers per animal were traced for both high and low SDH activity myofibers.

### Cytochrome c oxidase (COX) activity

COX enzyme activity was used as a measure of mitochondrial content as previously described^65^. Whole gastrocnemius (red) muscle tissue (~10 mg) was homogenized on ice in extraction buffer (0.1 M KH2PO4 and 2 mM EDTA pH 7.2) and enzyme activity was determined by the maximal oxidation rate of completely reduced cytochrome *c*, evaluated as a change in absorbance at 550 nm using an Eppendorf Biophotometer.

### Statistical Analysis

Data were analyzed using Graph Pad 6.0 software and values are reported as means ± standard error of the mean. Data were analyzed using Students t-test. Significance levels were set at p < 0.05.

## Results

### Gene targeting

Because the aspartate required for Pus1p activity is located in exon 3^66^, we targeted that region for disruption (see Fig. 1A). Nearly the entire exon was replaced in mouse embryonic stem cells with an internal ribosome entry site (IRES)/LacZ sequence and a PGK Neo cassette flanked by loxP sites (see Materials and Methods). The PGK Neo cassette was excised by breeding to CMV-CRE transgenic animals (Materials and Methods). To simplify the characterization and analysis of the knockout, and because it was already known that heterozygous parents and siblings of MLASA patients are unaffected^40^,^46^, only wild-type and homozygous mutant (*Pus1*^+/+^ and *Pus1*^−/−^, respectively) mice derived from heterozygous (*Pus1*^+/−^ *x Pus1*^+/−^) matings of animals backcrossed to C57BL/6J at least 5 generations, were used for experimental studies.

### Confirmation of Pus1p enzyme deficiency

**Figure 2:**
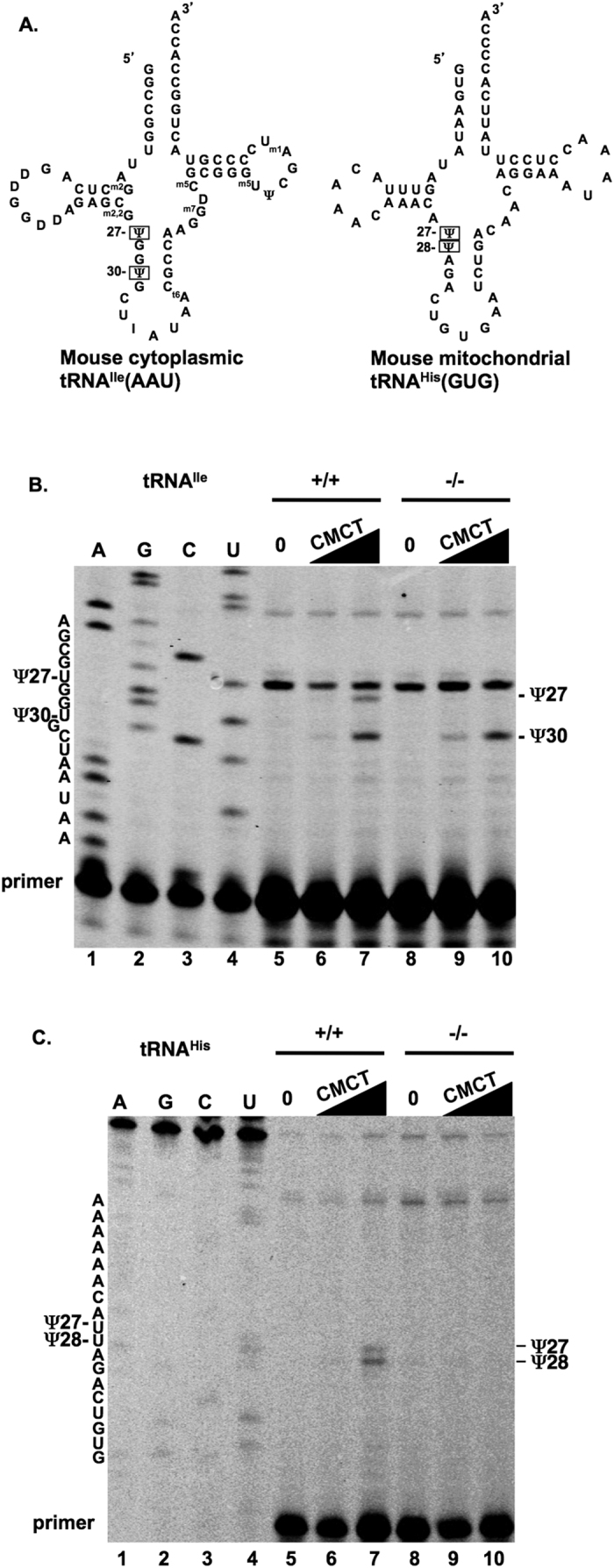
Primer extension assays to test for Pus1p activity. Panel (**A**) shows the sequence and predicted secondary structure^67^ of mouse cytoplasmic tRNA^Ile^(AAU) and mouse mitochondrial tRNA^His^(GUG). The known Ψs at positions 27 and 30 in tRNA^Ile^^67^ and predicted Ψs at positions 27 and 28 in tRNA^His^ are indicated by boxes. The additional modified nucleotides shown on tRNA^Ile^ are: D, dihydrouridine; I, inosine; ^m1^A, 1-methyladenine; ^m5^C, 5-methylcytidine; ^m5^U, 5-methyluridine; ^m7^G, 7-methylguanidine; ^m2,2^G, N2,N2-dimethylguanidine; ^m2^G, N2-methylguanidine; ^t6^A, N6-threonylcarbamoyladenine^71^. In panels (**B**,**C**) a fluorescently-labeled primer specific for mouse tRNA^Ile^ (**B**) and a primer specific for mouse tRNA^His^ (**C**) were was used in primer extension reactions to determine the location of Ψ in samples of mouse kidney total RNA as described in Materials and Methods. In panels (**B**,**C)** lanes 5–7 contain wild-type control RNA (0) or RNA samples reacted with 0.042 or 0.167M CMCT (solid triangle), whereas lanes 8–10 are the result when RNA from *Pus1*^−/−^ mice is used in the reactions. In panel (**B**) the sequence of tRNA^Ile^ in this region is shown in Lanes 1–4. In panel (**C**) the sequence of tRNA^His^ is shown in Lanes 1–4. For panels (**B**,**C**) the stops to RT that indicate Ψ in the primer extension reactions are shown on the right and positions of the modified uridines are indicated on the left, in the sequences. The band seen even in lanes not treated with CMCT, just below G26 in the sequence (lanes 5–10, panel B), is most likely due to the presence of N2,N2-dimethylguanidine (^m2,2^G, Fig. 2A) at position 26 in mouse cytoplasmic tRNA^Ile^(AAU)^71^. This nucleotide modification has been observed by others to be a stop to RT even in untreated RNA^72^.

The results from a real time, reverse transcriptase PCR analysis of three *Pus1*^+/+^ and three *Pus1*^−/−^ mice are shown in Fig. 1C. Samples from a real-time PCR protocol that employs one primer in the deleted exon, show the lack of a band for Pus1p mRNA in the *Pus1*^−/−^ samples. However, all the samples with cDNA have a band for the loading control, PPIA, illustrating that the *Pus1*^−/−^ mice lack mRNA that would code for an active enzyme. Pus1p modifies uridines at positions 27 and 28 in most cytoplasmic and mitochondrial tRNAs^18^,^27^,^28^,^66^ and position 30 in yeast and mouse pre-tRNA^Ile^(UAU)^28^,^29^. To determine if Pus1p activity was indeed absent in the *Pus1*^−/−^ mice, we assessed pseudouridinylation in cytoplasmic tRNA^Ile^(AAU) and mitochondrial tRNA^His^(GUG) (mt-tRNA^His^(GUG); see Fig. 2A) using a reverse transcriptase (RT) primer-extension/CMCT assay in total RNA from kidney tissue of wild-type and knockout mice (Fig. 2). This method exploits the fact that Ψ induces a stop in reverse transcription when reacted with CMCT. Positions 27 and 30 in mouse tRNA^Ile^ are pseudouridylated, but only position 27 is modified by Pus1p^28^,^29^,^67^. As expected, in RNA samples from *Pus1*^−/−^ mice we observed an absence of Ψ27 in tRNA^Ile^(AAU) (Fig. 2B) as well as Ψ27 and 28 in mt-tRNA^His^(GUG) (Fig. 2C). By contrast, also as expected, position 30 in tRNA^Ile^(AAU) was modified in the *Pus1*^−/−^ mice (Fig. 2B). Overall, these assays show that Pus1p activity is missing in the knockout mice and that there is no redundant activity that can modify these positions in the tRNAs.

### Pus1p deficiency phenotype

At seven weeks of age, female *Pus1*^−/−^ mice were significantly smaller than their wild-type littermates. The gastrocnemius to body weight ratio was also significantly smaller, indicating not only growth retardation, but also disproportionally decreased muscle mass (Table 1). Complete blood count (CBC) analysis demonstrated no differences in white blood cells (WBC), hemoglobin (HGB) or hematocrit (HCT), or in red blood cell (RBC) parameters, including RBC number (RBC), mean corpuscular volume (MCV), mean corpuscular hemoglobin (MCH), or red blood cell distribution width (RDW) (Table 2). Siderocytes and ringed sideroblasts were not present on iron-stained smears of peripheral blood and bone marrow, respectively (not shown). In sum, in contrast to MLASA patients, there appears to be no overt hematological phenotype in Pus1p deficient mice.

At 14 weeks of age, we re-examined body weight and muscle parameters in male and female *Pus1*^−/−^ mice. At this age, overall there was no significant difference in body weight for *Pus1*^−/−^ mice (Table 3), nor were there differences in the tibia length, or ratios for the tibialis anterior mass or gastrocnemius mass to body weight. We did observe significant differences in body weight and TA muscle mass between male wild-type and *Pus1*^−/−^ mice at this age. Wild-type male mouse body weight average was 29.4 ± 0.7 g and the male *Pus1*^−/−^ mice were 27.1 ± 0.6 g (p < 0.05) and the TA mass was 57.9 ± 1.5 for the wild type male mice and 50.8 ± 1.9 (p < 0.05) for the male *Pus1*^−/−^ mice but these differences in muscle mass disappeared when corrected for body weight. No significant differences were observed between female genotypes on body weight or muscle mass at this age. These data indicated that although body weight and muscle mass were delayed in the mutant compared to the wild type, these parameters equilibrated as the mice aged.

**Figure 3:**
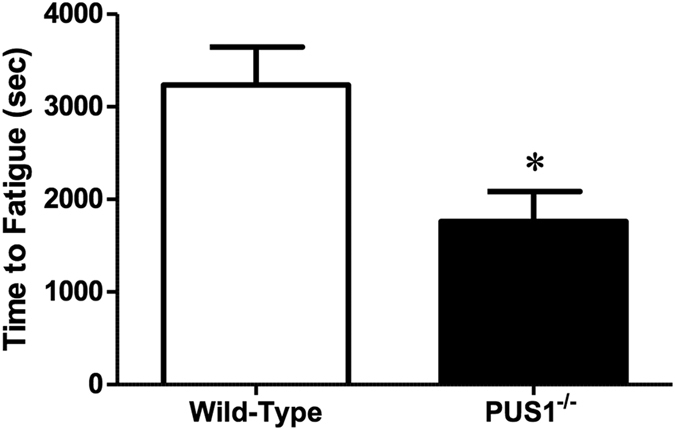
Endurance exercise capacity in wild-type and *Pus1*^−/−^ mice. Total time during a run to fatigue exercise test is presented in seconds (sec) in wild-type and *Pus1*^−/−^ mice. A total of 13 wild-type and 9 *Pus1*^−/−^ mice were examined. Data were analyzed with t-Tests and significance was set at p < 0.05. An asterisk (*) indicates the results for *Pus1*^−/−^ mice are significantly different from wild-type mice at p < 0.05.

To examine the effect of the loss of Pus1p on musculoskeletal function we assessed strength, neuromuscular coordination, and endurance capacity using standard functional tests. There were no significant differences in grip strength or in maximum rotorod speed and time (Table 4), suggesting that the loss of Pus1p does not have a direct effect on neuromuscular coordination. However, in an exercise treadmill test, *Pus1*^−/−^ mice fatigued more quickly exhibiting a 46% decrease in the run-to-fatigue time (p < 0.05, Fig. 3), suggesting an effect of the loss of Pus1p on endurance capacity.

**Figure 4:**
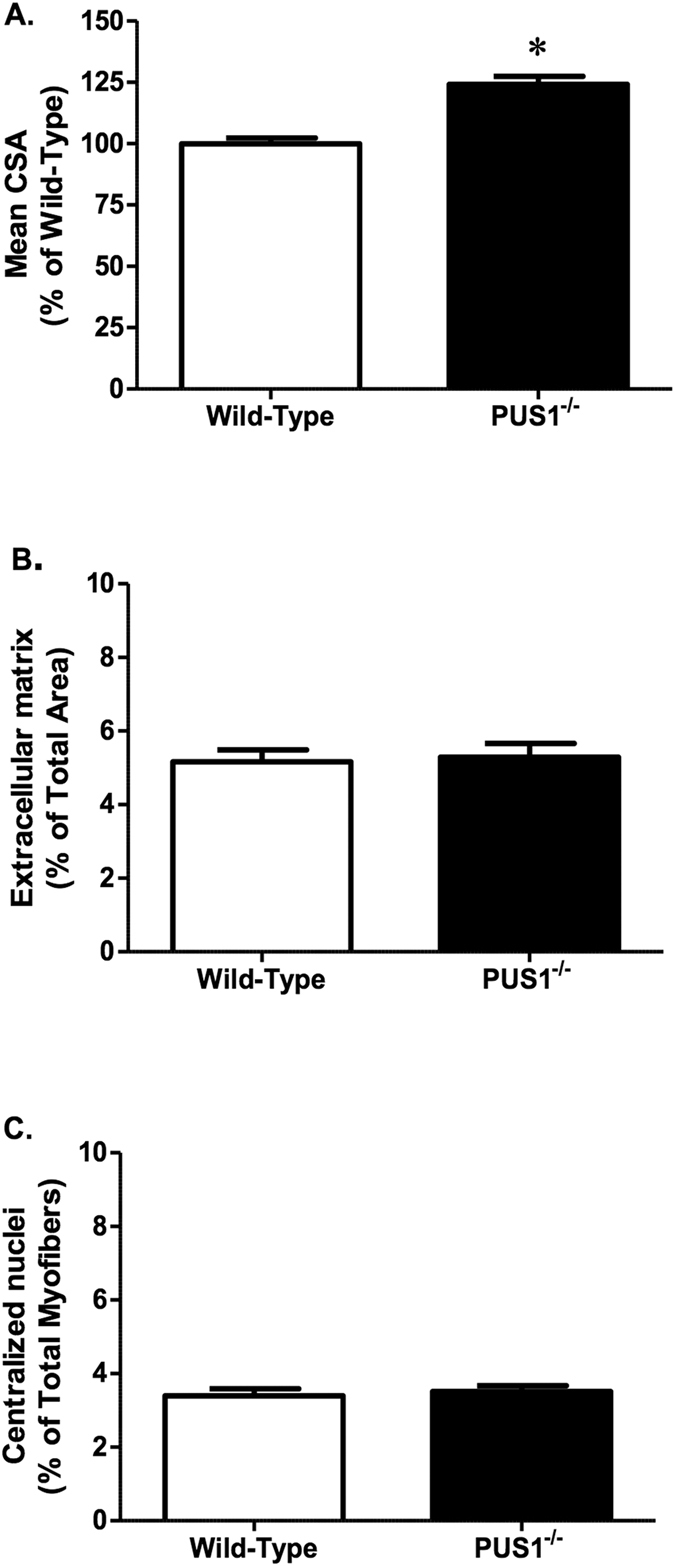
Tibialis anterior morphology in wild-type and *Pus1*^−/−^ mice. (**A**) Mean cross sectional area (CSA) of myofibers in the tibialis anterior in wild-type and *Pus1*^−/−^ mice. Data are expressed as the percentage of wild-type to account for differences in size between male and female mice. (**B**) The percentage of extracellular matrix area in the tibialis anterior in wild-type and *Pus1*^−/−^ mice. Data are expressed as the percentage of extracellular matrix area to the total myofiber area. (**C**) The percentage of centralized nuclei in the tibialis anterior in wild-type and *Pus1*^−/−^ mice. Data are expressed as the percentage of total myofibers containing centralized nuclei. Muscles from 13 wild-type and 9 *Pus1*^−/−^ were examined. Data were analyzed with t-Tests and significance was set at p < 0.05. An asterisk (*) indicates the results for *Pus1*^−/−^ mice are significantly different from wild-type mice at p < 0.05.

To examine the effect of the loss of Pus1p on muscle morphology, fiber type, and oxidative capacity, we performed quantitative morphometry on serial cross sections taken from the TA muscle. H&E stained muscle sections were used to examine general muscle morphology. There was a 24% increase in mean cross-sectional myofiber area in the tibialis anterior muscle in the *Pus1*^−/−^ mice compared to wild-type mice (Fig. 4A). However, there were no changes in the percentage of extracellular matrix area or centralized nuclei (Fig. 4B,C). Overall, this suggests the loss of Pus1p has an effect on skeletal muscle myofiber area that is not related to the degeneration and regeneration of muscle fibers.

**Figure 5:**
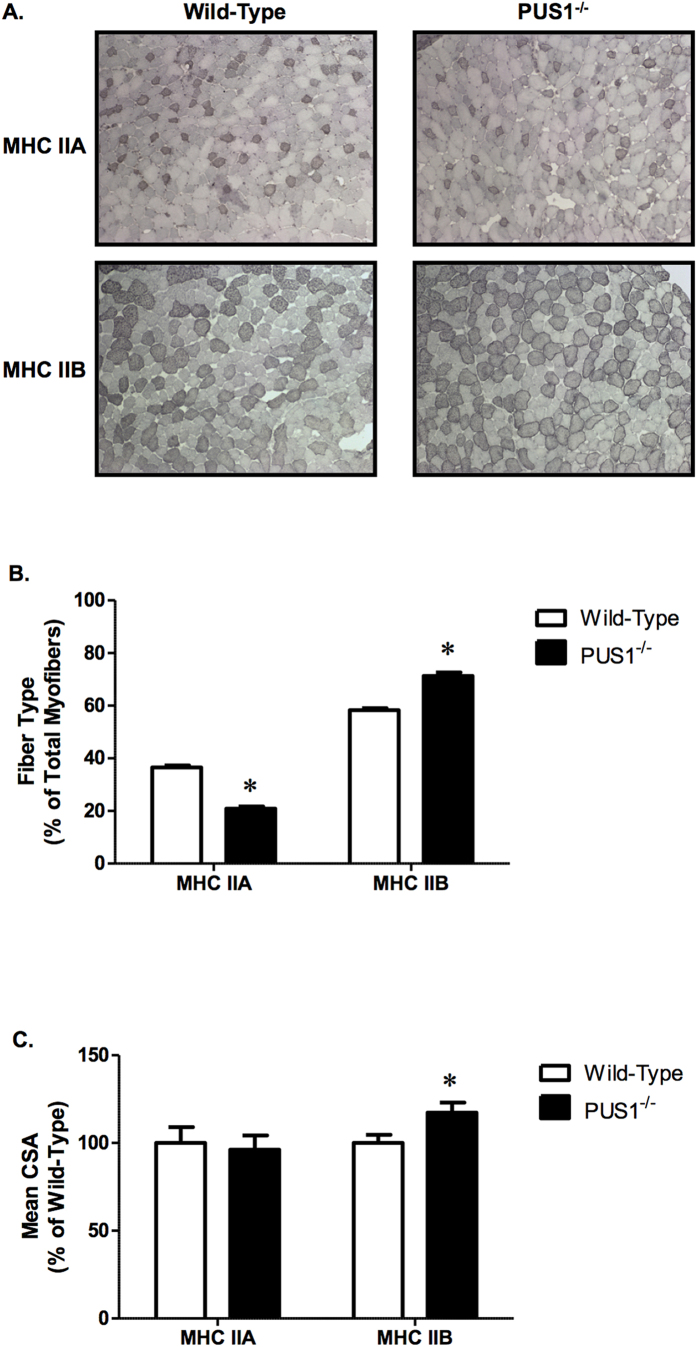
Tibialis anterior fiber type percentages and myofiber areas in wild-type and *Pus1*^−/−^ mice. (**A**) Representative images (10×) of tibialis anterior MHC IIA and IIB myofibers in wild-type and *Pus1*^−/−^ mice. (**B**) Percentage of MHC IIA and IIB myofibers in the tibialis anterior of wild-type and *Pus1*^−/−^ mice. Data are expressed as the percentage of total myofibers stained positive for MHC IIA and IIB. (**C**) Mean cross sectional area of MHC IIA and IIB myofibers in the tibialis anterior of wild-type and *Pus1*^−/−^ mice. Data are expressed as the percentage of wild-type to account for differences in size between male and female mice. Muscles from 13 wild-type and 9 *Pus1*^−/−^ were examined. Data were analyzed with t-Tests and significance was set at p < 0.05. An asterisk (*) indicates the results for *Pus1*^−/−^ mice are significantly different from wild-type mice at p < 0.05.

Muscle sample cross sections from the two genotypes were also stained with Gomori trichrome and NADH reductase stains (see Supplemental Figure S1) and the muscle from *Pus1*^−/−^ mice shows distinctly increased coarse red staining with Gomori Trichrome, and coarse reaction product with NADH reductase histochemistry. To further examine the myofiber changes, we examined the percentage and cross-sectional area of fibers stained positive for MHC type IIA and IIB. We found that the proportion of MHC IIA positive fibers decreased (37% versus 21%) and MHC IIB positive fibers reciprocally increased (58% versus 71%) in the *Pus1*^−/−^ mice (Fig. 5A,B). Interestingly while there was no change in the mean cross-sectional area of MHC IIA myofibers, there was a significant increase (17%) in the mean cross-sectional area of IIB myofibers in *Pus1*^−/−^ mice. This indicates that the histological changes in myofiber cross sectional area can be accounted for entirely by alterations in MHC IIB myofibers.

**Figure 6:**
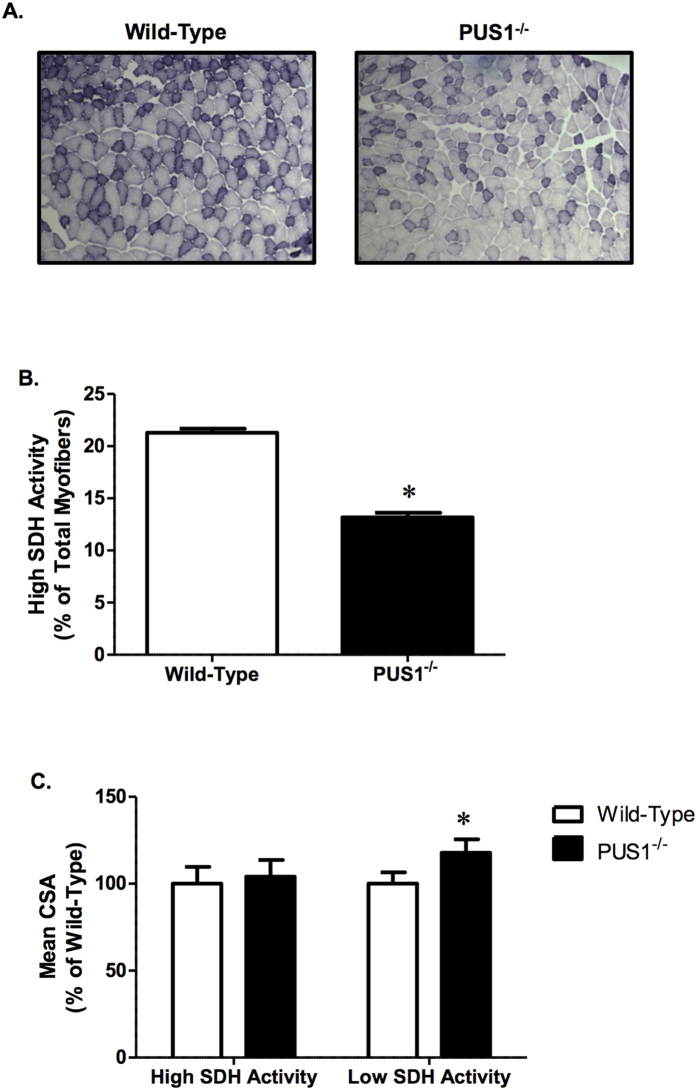
Tibialis anterior succinate dehydrogenase (SDH) activity in wild-type and *Pus1*^−/−^ mice. (**A**) Representative images (10×) of tibialis anterior SDH staining in wild-type and *Pus1*^−/−^ mice. (**B**) The percentage of high SDH activity myofibers in the tibialis anterior of wild-type and *Pus1*^−/−^ mice. (**C**) Mean cross sectional area of tibialis anterior high and low SDH activity myofibers in wild-type and *Pus1*^−/−^ mice. Data are expressed as the percentage of wild-type to account for differences in size between male and female mice. Muscles from 13 wild-type and 9 *Pus1*^−/−^ were examined. Data were analyzed with t-Tests and significance was set at p < 0.05. An asterisk (*) indicates the results for *Pus1*^−/−^ mice are significantly different from wild-type mice at p < 0.05.

To examine whether the absence of Pus1p has an effect on oxidative capacity, SDH staining was utilized to quantify the percentage and cross-sectional area of high SDH activity myofibers. There was a 38% decrease in the number of high SDH activity myofibers in the *Pus1*^−/−^ mice (Fig. 6A,B). Similar to the observations in the fiber typing (Fig. 5), there was no difference in the mean cross-sectional area of high SDH activity myofibers, but there was an increase in the mean cross-sectional area of low SDH activity myofibers (Fig. 6C). Additional analysis demonstrated that fibers staining for low SDH activity were also type IIB positive. *In toto*, these data support the conclusion that while there is an overall maintenance of muscle mass, there is a reduction in oxidative capacity that is related to lower high SDH activity myofibers and increased percentage and size of type IIB, glycolytic fibers.

Western blots of protein samples extracted from tissues (red and white gastrocnemius muscle, heart, liver, and brain) were probed with the Mitoprofile (Abcam, Inc.) cocktail of antibodies. This cocktail of five mono-clonal antibodies recognizes the most labile component of each of the five oxidative phosphorylation complexes, which can be used to determine differences in the levels of each complex between tissues from wild-type and *Pus1*^−/−^ mice. The only significant difference seen in all of the blots probed with Mitoprofile was in the heart, as Complex IV was elevated (41% higher; p < 0.05) in the *Pus1*^−/−^ mice (see Supplemental Figure S2). Given the SDH activity results presented above, we anticipated that the level of the band at 30 KDa, which is the Succinate dehydrogenase [ubiquinone] iron-sulfur subunit, mitochondrial (SDHB) protein, might be different between the two genotypes, but it was not.

**Figure 7:**
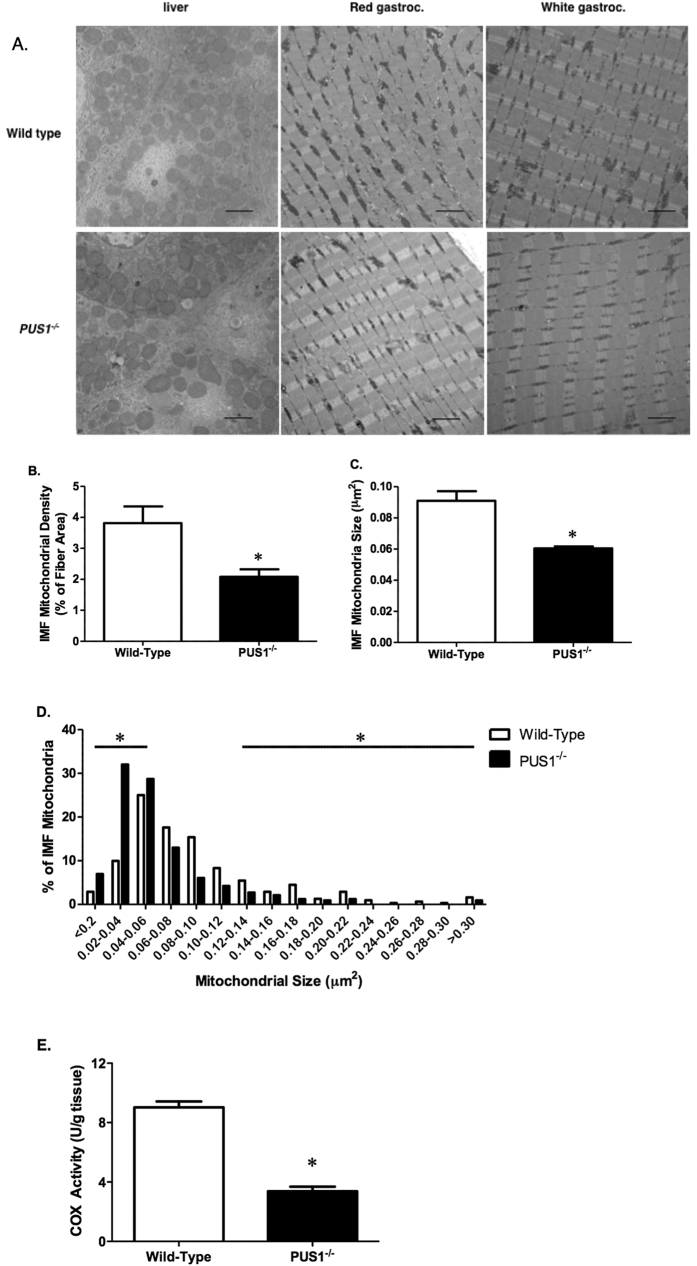
Transmission electron micrographs of liver and muscle samples in wild-type and *Pus1*^−/−^ mice. (**A**) Representative transmission electron micrographs (10,000X) of liver (*left column*), and the primarily red (*middle column*) and white portions (*right column*) of the gastrocnemius muscle from wild-type and *Pus1*^−/−^ mice. The scale bar on the photograph represents 2 microns. (**B**) The intermyofibrillar (IMF) mitochondrial density of the red portion of the gastrocnemius muscle in wild-type and *Pus1*^−/−^ mice. Data are presented as the percentage of mitochondrial area per total fiber area. (**C**) The IMF mitochondria size (μm^2^) of the red portion of the gastrocnemius muscle in wild-type and *Pus1*^−/−^ mice. Data are presented as the total mitochondrial size divided by the total fiber area. (**D**) The IMF mitochondria size (μm^2^) distribution of red gastrocnemius muscle in wild-type and *Pus1*^−/−^ mice. Muscles from 3 animals per group were examined (~100 IMF mitochondria per animal). An asterisk (*) indicates the results for *Pus1*^−/−^ mice are significantly different from wild-type mice at p < 0.05. (**E**) Cytochrome c oxidase (COX) activity of the red portion of the gastrocnemius muscle in wild-type and *Pus1*^−/−^ mice. An asterisk (*) indicates the results for *Pus1*^−/−^ mice are significantly different from wild-type mice at p < 0.05.

Transmission electron microscopy of red and white gastrocnemius muscle from the wild-type and *Pus1*^−/−^ mice confirmed, as suggested by histochemical studies, that skeletal muscle mitochondria are affected by the loss of Pus1p. As shown in Fig. 7A, the mitochondria in the *Pus1*^−/−^ animals appear smaller and less abundant than those in the muscles from wild-type mice. None of the mitochondria, even those in the subsarcolemmal regions, showed evidence of paracrystalline inclusion bodies similar to those seen muscle biopsies from MLASA patients^36^,^39^. Unlike skeletal muscle mitochondria, liver mitochondria in *Pus1*^−/−^ mice are not ultrastructurally abnormal (Fig. 7A). These differences in muscle mitochondria are manifest as a 45% reduction in mitochondrial density (p < 0.05, Fig. 7B) and a 33% reduction in average mitochondrial size (p < 0.05, Fig. 7C). There is an increase in the number of very small mitochondria in the *Pus1*^−/−^ mice (Fig. 7D). COX activity was also measured in crude homogenates of the red gastrocnemius muscle to provide an assessment of mitochondrial function. *Pus1*^−/−^ mice demonstrated a 63% reduction in COX activity compared to wild-type mice (p < 0.0001; Fig. 7E).

The results from microarray analysis (see Methods for experimental specifics) from six tissues (brain, heart, kidney, liver, red gastrocnemius muscle, and white gastrocnemius muscle) of eight mice (four wild-type and four *Pus1*^−/−^ mice, two males and two females of each genotype) show patterns that correlate with the above results. Leading Edge Analysis shows that several pathways, such as those involved in mitochondrial biogenesis, respiration, and oxidative phosphorylation, for example, are affected in all the tissues tested (see Table S1 in Supplemental Material). Many of the genes listed in the table are enriched in the tissues from *Pus1*^−/−^ mice when compared with the tissues from wild-type mice (see enrichment scores in columns for brain, heart, kidney, liver, and to some extent, red muscle in Table S1). But in white skeletal muscle the levels of many of those genes are enriched in the wild-type mice relative to the *Pus1*^−/−^ mice. In other words, the *Pus1*^−/−^ mice appear to be compensating for the loss of Pus1p by increasing the expression of genes involved in mitochondrial biogenesis and respiration in brain, heart, kidney, liver, and to some extent in red skeletal muscle, but not in white skeletal muscle. This predicts that white skeletal muscle will be the most affected in these pathways, which supports the histological and biochemical analysis outlined above. The top 50 features from the Gene Set Enrichment Analysis that are enriched in the tissues from wild-type mice and enriched in the *Pus1*^−/−^ mice, are given in heat maps in Supplemental Figure S3A–F. For each tissue these are the 50 most enriched genes in the two genotypes of mice. An interesting feature in the heat map for Brain samples (Figure S3A) for instance, is that there are five aminoacyl tRNA-synthetases that are enriched in the *Pus1*^−/−^ mouse brain, significant because the other cause for MLASA is the mutation of an aminoacyl tRNA-synthetase (see Introduction).

Taken collectively, these data demonstrate that Pus1p deficiency alters mouse skeletal muscle metabolism related to mitochondrial content and oxidative capacity, and these alterations may account for impaired exercise capacity independent of changes in muscle mass.

## Discussion

MLASA is caused by mutations of either the *Pus1* or the *YARS2* genes, encoding Pus1p^40^,^41^ or mitochondrial tyrosyl-tRNA synthetase^42^,^43^,^44^,^45^, respectively. It is relatively straight forward to understand how the reduction in charging efficiency of an amino acyl-tRNA synthetase (ARS) found in mitochondria, combined with a lower level of expression of ARS in skeletal muscle, would negatively impact mitochondrial protein synthesis and lead to a oxidative phosphorylation disorder such as MLASA^42^. It is more difficult to grasp why the loss of Pus1p, an enzyme that forms Ψ on most cytoplasmic as well as the majority of mitochondrial tRNAs^46^, would result in the MLASA phenotype, affecting primarily skeletal muscle and bone marrow^38^. The *Pus1* knockout mouse model described will help us answer this question in ways that MLASA patient-derived cell lines cannot^46^,^47^. In addition, the mouse model will provide a way to test interventions that will benefit MLASA patients.

Lacking a full complement of modifications, the tRNAs found in *Pus1*^−/−^ mice will probably be affected in a number of ways. Transport of tRNAs out of the nucleus may be compromised, as was seen with yeast *Pus1*/*Pus4* double mutants^48^. In preliminary experiments, RNA samples isolated from tissues of wild-type and *Pus1*^−/−^ mice have been hybridized to tRNA microarrays^68^, and the results indicate that the levels of certain tRNAs are modulated in the *Pus1*^−/−^ mice (unpublished results). *In situ* hybridization analysis of affected tRNAs in tissue samples from the knockout mice will be instrumental in determining if these tRNAs are retained in the nucleus, as the yeast evidence would suggest.

With regard to the differences seen in the muscle fibers, the TA muscle of the *Pus1*^−/−^ mice showed an increase in the percentage of fibers expressing myosin heavy chain IIB and a decrease in IIA positive fibers relative to wild-type muscle. The overall increase in the mean cross sectional area in the muscles from the *Pus1*^−/−^ mice is highly influenced by the increased percentage of large Type IIB fibers. This is indicative of a shift to a faster, more glycolytic muscle phenotype and would be expected when one considers the characteristics of MLASA patients: exercise intolerance and elevated levels of lactic acid. The timing and causes for these shifts in fiber composition are not known, but given exercise intolerance was seen in the *Pus1*^−/−^ mice, we will be able to begin to understand the processes involved with this model of MLASA.

The decrease in COX activity and the percentage of high SDH activity fibers in the TA muscle in the *Pus1*^−/−^ mice when compared to wild-type mice is indicative of a reduction in oxidative capacity in the muscles of the knockout mice. SDH is part of complex II and MLASA case studies have not reported irregularities with complex II from muscle biopsies but they have noted deficiencies in complexes I and IV^38^,^39^,^41^ in samples from MLASA patients. With this new model, it will now be possible to isolate mitochondria from fast and slow muscle types as well as other tissues, such as heart, liver and brain, for comparison of inner membrane complexes and respiration capacity.

Why is there no sign of anemia in the *Pus1*^−/−^ mice? In published case reports of MLASA patients, the age where anemia is diagnosed is variable. In one report of two brothers, both were in their late teens (18 and 19) before anemia was added to the myopathy diagnosis^36^. Inbal *et al*.^37^ reported that a sister and brother were diagnosed with anemia at 14 and 10 respectively. Additional patients with MLASA and with the same ethnic background^38^,^39^, were diagnosed with anemia at 8, 12, 17, and 19 years of age, with an additional patient requiring transfusions by 16 years old due to severe anemia (this patient was at least 11 years old before anemia diagnosis). In these published cases, exercise intolerance is reported as an initiating symptom and anemia is either present initially or added to the diagnosis subsequently. The severity of the sideroblastic anemia fluctuated in two of the described cases^36^. It is possible that the mice will eventually develop anemia as they age. However, it is equally probable that due to differences in mitochondrial iron metabolism in mice and humans, including the absence of ring sideroblasts in most mouse models of sideroblastic anemia, that mice are simply not susceptible to this disorder^69^,^70^.

Overall, these results demonstrate that there are alterations in muscle metabolism related to mitochondrial content and oxidative capacity in the *Pus1*^−/−^ mice. The *Pus1* knockout model will eventually be used to test interventions to alleviate the symptoms of MLASA. The efficacy of a number of pharmaceuticals that are in clinical trials could be tested in this model and perhaps yield a treatment for this devastating disease.

## Additional Information

**How to cite this article**: Mangum, J. E. *et al*. Pseudouridine synthase 1 deficient mice, a model for Mitochondrial Myopathy with Sideroblastic Anemia, exhibit muscle morphology and physiology alterations. *Sci. Rep.* **6**, 26202; doi: 10.1038/srep26202 (2016).

## Supplementary Material

Supplementary Information

## Figures and Tables

**Table 1:** Body weight and relative gastrocnemius muscle mass in female wild-type and *Pus1* ^−/−^ mice at 7 weeks of age.

	**n**	**Body weight (BW;g)**^1^	**Gastroc/BW (×100)**
WT	7	19.4 ± 0.3	0.54 ± 0.01
*Pus1*^−/−^	5	15.8 ± 0.6†	0.48 ± 0.02*

Abbreviations: n, sample size. BW, body weight. g, grams. Gastroc, gastrocnemius.

^1^Entries are given as the mean plus/minus the standard error of the mean.

^†^Significant difference in the means of the two genotypes, p < 0.01.

^*^Significant difference in the means of the two genotypes, p < 0.05.

**Table 2:** Blood parameters in female wild-type and *Pus1* ^−/−^ mice at 7 weeks of age.

	**n**	**WBC ×10**^**3**^**/μl**^1^	**RBC ×10**^**−3**^**/μl**	**HGB g/dL**	**HCT%**	**MCV fL**	**MCH pg**	**RDW%**
WT	6	6.05 ± 0.31	8.27 ± 0.23	13.0 ± 0.2	49.3 ± 1.5	59.6 ± 0.3	15.8 ± 0.3	12.6 ± 0.2
*Pus1*^−/−^	4	6.12 ± 1.67	8.13 ± 0.25	12.9 ± 0.3	47.0 ± 1.7	57.9 ± 0.9	16.0 ± 0.2	12.8 ± 0.3

Abbreviations: WT, wild-type. WBC, white blood cell. RBC, red blood cell. HGB, hemoglobin. HCT, hematocrit. MCV, mean corpuscular volume. MCH, mean corpuscular hemoglobin. RDW, Red blood cell distribution width. μl, microliter, g/dL. gram per deciliter. %, percent. fL, femtoliters (×10^−15^ L). pg, picogram.

^1^Entries are given as the mean plus/minus the standard error of the mean.

**Table 3:** Body weight, tibia length and muscle mass to body weight ratios in wild-type and *Pus1* ^−/−^ mice at 14 weeks of age.

	n*	**Body weight (BW; g)**^1^	**Tibia Length (mm)**	**TA mass/BW (×100)**	**Gastroc Mass/BW (×100)**
WT	13	25.8 ± 1.4	16.8 ± 0.1	0.19 ± 0.004	0.51 ± 0.01
*Pus1*^−/−^	9	23.5 ± 1.4	16.4 ± 0.2	0.18 ± 0.004	0.50 ± 0.01

Abbreviations: n, sample size. WT, wild-type. BW, body weight. g, grams. mm, millimeter. TA, tibialis anterior. Gastroc, gastrocnemius.

^1^Entries are given as the mean ± standard error of the mean.

^*^For WT there were 8 males and 5 females, for *Pus1*^−/−^ there were 4 males and 5 females. A body weight for a female *Pus1*^−/−^ mouse was not taken at sacrifice (n = 1); therefore, data for 8 mice are presented in Table 3. A total of 9 mice were used throughout all other measures.

**Table 4:** Average grip strength and max rotorod speed and time in male and female wild-type and *Pus1* ^−/−^ mice.

	**n**^1^	**Grip Strength (N)**	**Max Rotorod Speed (rpm)**	**Max Rotorod Time (sec)**
WT	13	2.0 ± 0.1	19.8 ± 1.2	111.8 ± 24.3
*Pus1*^−/−^	9	1.8 ± 0.1	20.6 ± 1.4	107.5 ± 26.0

Abbreviations: n, sample size. WT, wild-type. N, Newton. rpm, revolutions per minute. sec, seconds.

^1^Entries are given as the mean plus/minus the standard error of the mean. For WT there were 8 males and 5 females and for *Pus1*^−/−^ there were 4 males and 5 females.
