# Supplementary material for: Pseudouridine synthase 1 deficient mice, a model for Mitochondrial Myopathy with Sideroblastic Anemia, exhibit muscle morphology and physiology alterations
Source: Sci Rep. 2016 May 20;6:26202. doi: 10.1038/srep26202 (PMC4873756; doi:10.1038/srep26202)
Supplement: Supplementary Information [file srep26202-s1.pdf]

Pseudouridine synthase 1 deficient mice, a model for Mitochondrial Myopathy with Sideroblastic Anemia (MLASA), exhibit muscle morphology and physiology alterations.

Joshua E. Mangum[1], Justin P. Hardee[1], Dennis K. Fix[1], Melissa J. Puppa[1], Johnathon Elkes[2], Diego Altomare[3], Yelena Bykhovskaya[4], Dean R. Campagna[5], Paul J. Schmidt[5], Anoop K. Sendamarai[5], Hart G. W. Lidov[5], Shayne Barlow[6], Nathan Fischel-Ghodsian[4], Mark D. Fleming[5], James A. Carson[1], and Jeffrey R. Patton[2*]

Supplemental Table S1
Values from Leading Edge Analysis Heat Map for selected genes from mouse tissues.

Genes	Brain[2]	Heart	Kidney	Liver	Skeletal Muscle (red)	Skeletal Muscle (white)
NADH:ubiquinone oxidoreductase core subunit 1 (ND1)	0.242	-	0.213	0.703	0.675	-0.326
Cytochrome oxidase 2 (Cox 2)	-	-	-	1.230	-	-
PGC-1α[1]	-	-	0.185	-	0.325	-0.441
Nuclear respiratory factor 1 (NRF1)	-	-	-0.521	-	0.546	-0.389
SDHB	-	-	0.240	0.533	-	-0.673
TFAM	-	0.179	0.739	-	-	0.395
Mitofusin 1 (Mfn1)	-	-	0.251	-	-	-0.475
Mitofusin 2 (Mfn2)	-	-	-	-	-	-0.673
NADH:ubiquinone oxidoreductase core subunit 1α subcomplex 1 (NDUFA1)	-	-	0.387	0.390	0.347	-0.424
NDUFA2	0.229	-	0.172	0.191	0.356	-0.250
NDUFA5	0.565	-	-	-	-	-0.380
NDUFA9	-	-	0.193	-	-	-0.387
NDUFA10	-	-	-	0.697	-	-0.465
NDUFA12	-	-	-	0.544	-	-
NDUFA13	0.403	-	-	0.682	-	-0.471
NDUFAB1 (α/β subcomplex 1)	-	0.802	0.766	0.945	-	0.302
NDUFB3 (β subcomplex 3)	0.672	0.487	0.306	0.312	-	-1.213
NDUFB4	0.674	-	-	1.163	-	-
NDUFB5	-	-	0.463	0.337	-	-0.735
NDUFB6	-	-	0.332	0.419	-	-
NDUFB9	-	-	-	-	-	-0.425
NDUFB10	0.364	-	-	0.236	-	-0.812
NDUFBS1 (Fe-S protein 1)	0.803	-	0.604	-	0.443	-0.987
NDUFBS2	-	-	0.549	0.392	-	-
NDUFBS3	-	-	0.445	-	0.477	-0.829
NDUFBS4	-	-	0.512	0.439	0.645	-
NDUFBS6	0.317	-	0.327	0.771	-	-0.476
NDUFBS7	-	0.189	0.172	0.232	-	-0.446
NDUFBS8	0.222	-	0.231	0.788	-	-
NDUFV1 (Flavoprotein 1)	-	-	1.127	0.834	-	-0.614
NDUFV2	0.861	-	-	0.240	-	-0.400

[1]Abreviations in this column are: PGC-1α, Peroxisome proliferator-activated receptor gamma coactivator-1 alpha, also known as PPARGC1a; SDHB, succinate dehydrogenase (ubiquinone) iron-sulfur subunit, mitochondrial; TFAM, Transcription factor A,

mitochondrial. For the NDUF subunits and proteins, the first subunit for that group is named and is in parentheses, the rest of the protein names can be inferred.

[2]The Enrichment Scores (ES) are used to determine the position of the gene in a list of ranked genes when comparing the expression levels between total RNA from samples from wild-type and *Pus1*$^{-/-}$ mouse tissues. A positive ES indicates gene enrichment in the *Pus1*$^{-/-}$ mice, a negative ES (red font) indicates enrichment in samples from the wild-type mice. A dash (-) in the box indicates that gene did not appear (no enrichment in either genotype) in the Leading Edge Analysis for that tissue.

Supplemental Figure Legends

Figure S1. Gomori trichrome stain and NADH reductase histochemistry stain of muscle fibers. These are representative samples of skeletal muscle from wild-type and *Pus1*$^{-/-}$ mice (females) where the samples have been stained with Gomori trichrome stain or NADH tetrazolium reductase. All images were taken at 60X magnification.

Figure S2. Representative Western blot of proteins samples from mouse heart tissue probed with Mitoprofile antibody cocktail. Protein samples were extracted from heart tissue from four wild-type and four *Pus1*$^{-/-}$ mice, electrophoresed on a gel, transferred to a PVDF membrane and incubated with the Mitoprofile antibody cocktail as the primary antibody and LiCor IRDye 800 goat anti-mouse secondary antibody as described in Materials and Methods. The blot was then scanned in dual colors with a LiCor Odyssey CLx instrument. Lane 1 is the Li-Cor Chameleon Duo marker, with sizes in kDa shown on the left. Lanes 2 through 5 are samples from four wild-type mice. Lanes 6 through 9 are samples from four *Pus1*$^{-/-}$ mice.

Figure S3. GSEA Heat Map of top 50 features for tissues from wild-type and *Pus1*$^{-/-}$ mice. The Gene Set Enrichment Analysis Heat Maps for the DNA Microarrays described in the Methods section are shown for each tissue. There were four samples for each genotype and in the left-upper corner, *Pus1*$^{-/-}$ mouse samples are indicated by grey hatching and the wild-type samples are indicated by yellow hatching. The heat maps for the samples for each of the tissues are in the panels: (A) brain, (B) heart, (C) kidney, (D)

liver, (E) skeletal muscle, gastrocnemius, red, slow, and (F) skeletal muscle, gastrocnemius, white, fast.

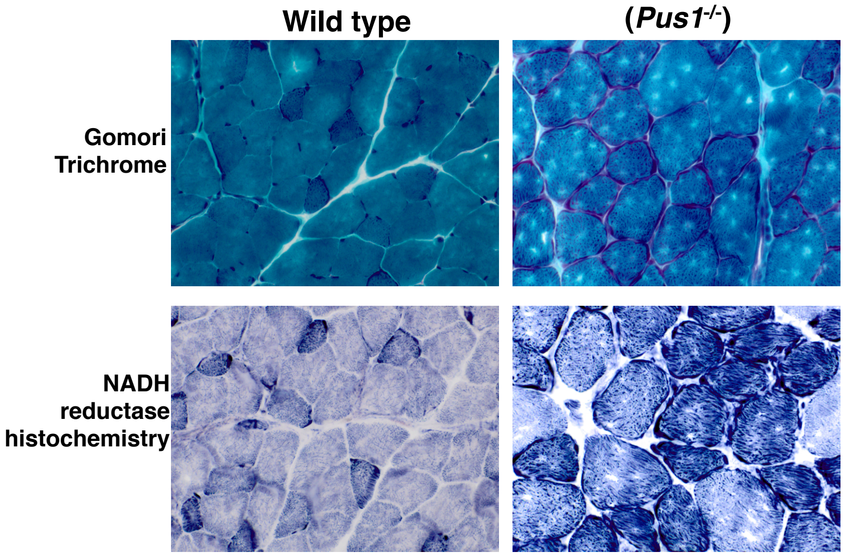
Wild type
(Pus1-/-)
Gomori Trichrome
NADH reductase histochemistry

Figure S1

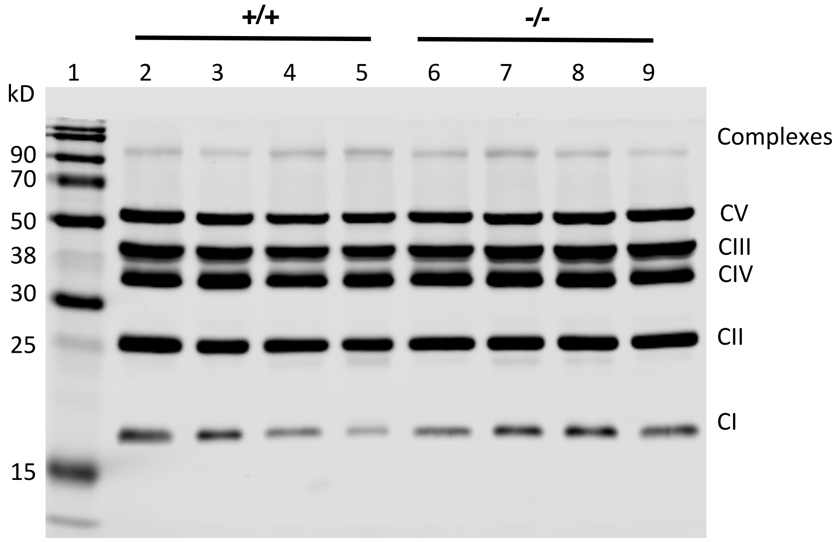

Figure S2

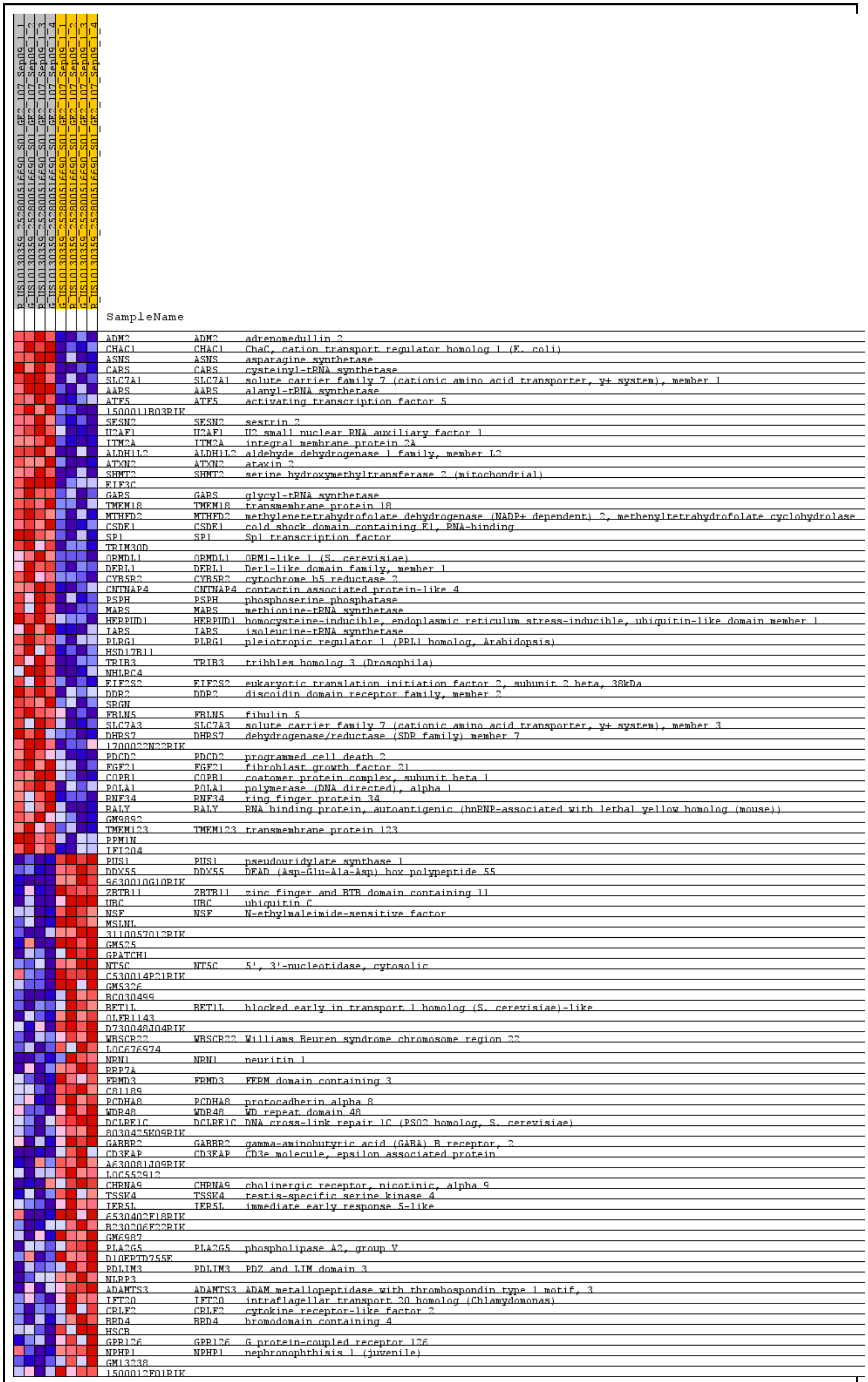

Fig 1: heat_map
Heat Map of the top 50 features for each phenotype in BRAIN-Normalized FLAG Filtered Data_collapsed_to_symbols

Figure S3A

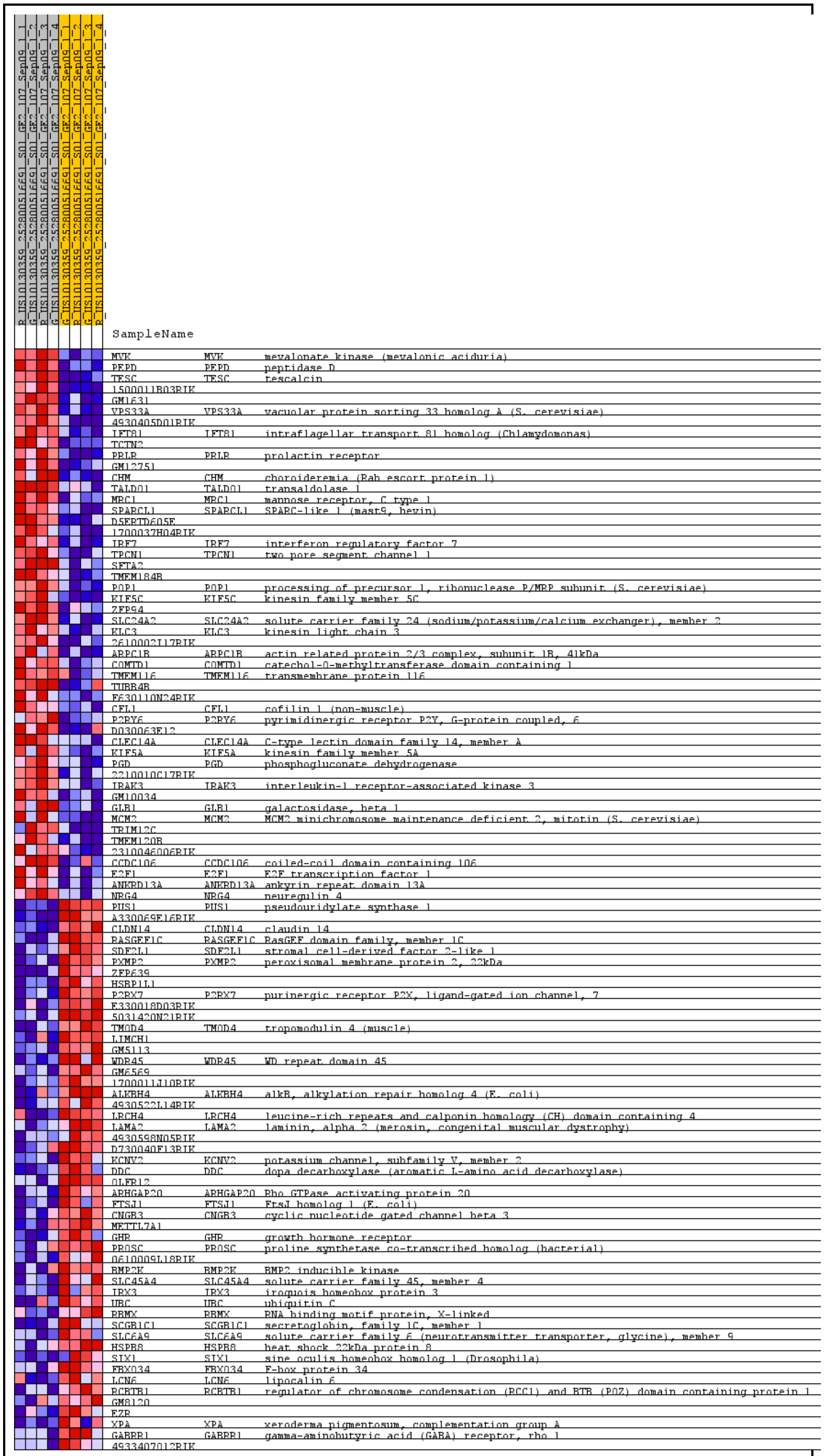

Fig 1: heat_map
Heat Map of the top 50 features for each phenotype in HEART-Normalized FLAG Filtered Data_collapsed_to_symbols

Figure S3B

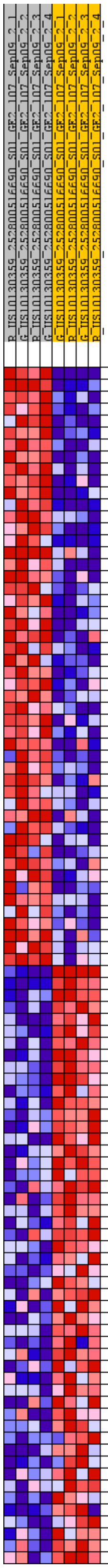

SampleName		
VPS33A	VPS33A	vacuolar protein sorting 33 homolog A (S. cerevisiae)
ASB11	ASB11	ankyrin repeat and SOCS box-containing 11
TMEM116	TMEM116	transmembrane protein 116
HOXB7	HOXB7	homeobox B7
CDC20	CDC20	CDC20 cell division cycle 20 homolog (S. cerevisiae)
SLC25A12	SLC25A12	solute carrier family 25 (mitochondrial carrier, Aralar), member 12
CTSH	CTSH	cathepsin H
PGK1	PGK1	phosphoglycerate kinase 1
MVK	MVK	mevalonate kinase (mevalonic aciduria)
NUP85	NUP85	nucleoporin 85kDa
A930038B10RIK		
CCNA2	CCNA2	cyclin A2
SPACA7		
KDM2B		
PARP2	PARP2	poly (ADP-ribose) polymerase family, member 2
SLC8A1	SLC8A1	solute carrier family 8 (sodium/calcium exchanger), member 1
XRCC6BP1	XRCC6BP1	XRCC6 binding protein 1
MSRA	MSRA	methionine sulfoxide reductase A
SIRT4	SIRT4	sirtuin (silent mating type information regulation 2 homolog) 4 (S. cerevisiae)
OPTN	OPTN	optineurin
GM5537		
TMEM140	TMEM140	transmembrane protein 140
TTF2	TTF2	transcription termination factor, RNA polymerase II
CDS1	CDS1	CDP-diacylglycerol synthase (phosphatidate cytidylyltransferase) 1
ACAA1A		
STMN1	STMN1	stathmin 1/oncoprotein 18
PAPPA	PAPPA	pregnancy-associated plasma protein A, pappalysin 1
PHLPP2		
1500011B03RIK		
CLCA2	CLCA2	chloride channel, calcium activated, family member 2
PSMC1	PSMC1	proteasome (prosome, macropain) 26S subunit, ATPase, 1
SEMA5B	SEMA5B	sema domain, seven thrombospondin repeats (type 1 and type 1-like), transmembrane domain (TM) and short cytoplasmic domain, (semaphorin) 5B
BIRC5	BIRC5	baculoviral IAP repeat-containing 5 (survivin)
POP1	POP1	processing of precursor 1, ribonuclease P/MRP subunit (S. cerevisiae)
GM11223		
4921513D23RIK		
CDCA5	CDCA5	cell division cycle associated 5
RNASEL	RNASEL	ribonuclease L (2',5'-oligoisoadenylate synthetase-dependent)
CCNB2	CCNB2	cyclin B2
FAF1	FAF1	Fas (TNFRSF6) associated factor 1
DNASE1	DNASE1	deoxyribonuclease I
TPX2	TPX2	TPX2, microtubule-associated, homolog (Xenopus laevis)
IFT80	IFT80	intraflagellar transport 80 homolog (Chlamydomonas)
FBXO9	FBXO9	F-box protein 9
GM6524		
RILP	RILP	Rab interacting lysosomal protein
SVIP		
ZMYND10	ZMYND10	zinc finger, MYND-type containing 10
TEC	TEC	tec protein tyrosine kinase
SECTM1A		
PUS1	PUS1	pseudouridylate synthase 1
4930522L14RIK		
LOC638517		
UBC	UBC	ubiquitin C
TESC	TESC	tescalcin
HSCB		
AGPAT1	AGPAT1	1-acylglycerol-3-phosphate O-acyltransferase 1 (lysophosphatidic acid acyltransferase, alpha)
FYTTD1	FYTTD1	forty-two-three domain containing 1
RNF4	RNF4	ring finger protein 4
NPY6R	NPY6R	neuropeptide Y receptor Y6 (pseudogene)
PVRL4	PVRL4	poliovirus receptor-related 4
POR	POR	P450 (cytochrome) oxidoreductase
KCTD15	KCTD15	potassium channel tetramerisation domain containing 15
CRLF2	CRLF2	cytokine receptor-like factor 2
GTF2I	GTF2I	general transcription factor II, i
DNAJB4	DNAJB4	DnaJ (Hsp40) homolog, subfamily B, member 4
TST	TST	thiosulfate sulfurtransferase (rhodanese)
SMARCB1	SMARCB1	SWI/SNF related, matrix associated, actin dependent regulator of chromatin, subfamily b, member 1
BC048609		
PLLP	PLLP	plasma membrane proteolipid (plasmolipin)
KIT	KIT	v-kit Hardy-Zuckerman 4 feline sarcoma viral oncogene homolog
TESK1	TESK1	testis-specific kinase 1
LOC100046456		
DYNLL1	DYNLL1	dynein, light chain, LC8-type 1
DNAJC5	DNAJC5	DnaJ (Hsp40) homolog, subfamily C, member 5
RAC1	RAC1	ras-related C3 botulinum toxin substrate 1 (rho family, small GTP binding protein Rac1)
LRRC49	LRRC49	leucine rich repeat containing 49
STBD1		
GANAB	GANAB	glucosidase, alpha; neutral AB
GRB10	GRB10	growth factor receptor-bound protein 10
CIT	CIT	citron (rho-interacting, serine/threonine kinase 21)
HIPK1	HIPK1	homeodomain interacting protein kinase 1
TSPAN31	TSPAN31	tetraspanin 31
RRP7A		
GPR64	GPR64	G protein-coupled receptor 64
CASC3	CASC3	cancer susceptibility candidate 3
INO80B		
CUX2		
TMEM39A	TMEM39A	transmembrane protein 39A
ETNK2	ETNK2	ethanolamine kinase 2
SCTR	SCTR	secretin receptor
NLRC3		
FAT1		
GM4349		
2310067B10RIK		
FOLR4		
CAPS2	CAPS2	calcyphosine 2
CRTC1	CRTC1	CREB regulated transcription coactivator 1
RALB	RALB	v-ral simian leukemia viral oncogene homolog B (ras related; GTP binding protein)
RRS1	RRS1	RRS1 ribosome biogenesis regulator homolog (S. cerevisiae)

Fig 1: heat_map
Heat Map of the top 50 features for each phenotype in KIDNEY-Normalized FLAG Filtered Data_collapsed_to_symbols

Figure S3C

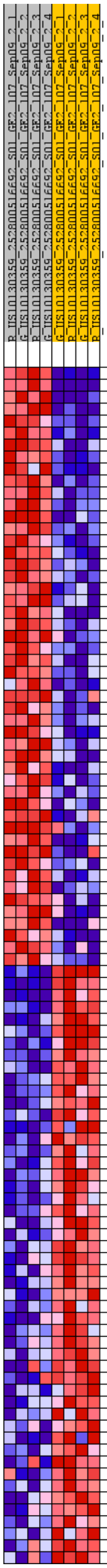

SampleName

PEBP1	PEBP1	phosphatidylethanolamine binding protein 1
SHCBP1	SHCBP1	SHC SH2-domain binding protein 1
FRMD4A	FRMD4A	FERM domain containing 4A
TPX2	TPX2	TPX2, microtubule-associated, homolog (Xenopus laevis)
MCM3	MCM3	MCM3 minichromosome maintenance deficient 3 (S. cerevisiae)
GYLTL1B	GYLTL1B	glycosyltransferase-like 1B
DNALC4		
GM6788		
HSD17B11		
MLKL	MLKL	mixed lineage kinase domain-like
TUBB6	TUBB6	tubulin, beta 6
DNAJC9	DNAJC9	DnaJ (Hsp40) homolog, subfamily C, member 9
L3MBTL2	L3MBTL2	l(3)mbt-like 2 (Drosophila)
CENPA	CENPA	centromere protein A
MCM2	MCM2	MCM2 minichromosome maintenance deficient 2, mitotin (S. cerevisiae)
RNF26	RNF26	ring finger protein 26
VAC14	VAC14	Vac14 homolog (S. cerevisiae)
1500011B03RIK		
CYP2C55		
GSTM3	GSTM3	glutathione S-transferase M3 (brain)
GATC		
DNAJC18	DNAJC18	DnaJ (Hsp40) homolog, subfamily C, member 18
VPS33A	VPS33A	vacuolar protein sorting 33 homolog A (S. cerevisiae)
MDC1	MDC1	mediator of DNA damage checkpoint 1
IFITM6		
NAB2	NAB2	NGFI-A binding protein 2 (EGR1 binding protein 2)
ECT2	ECT2	epithelial cell transforming sequence 2 oncogene
RAB37	RAB37	RAB37, member RAS oncogene family
COQ5	COQ5	coenzyme Q5 homolog, methyltransferase (S. cerevisiae)
TRIM59	TRIM59	tripartite motif-containing 59
NLRX1		
IFT81	IFT81	intraflagellar transport 81 homolog (Chlamydomonas)
ANLN	ANLN	anillin, actin binding protein
OAS1F		
H2-Q8		
H2-T3		
HPS1	HPS1	Hermansky-Pudlak syndrome 1
TRIP12	TRIP12	thyroid hormone receptor interactor 12
PI16	PI16	peptidase inhibitor 16
AURKA	AURKA	aurora kinase A
GM8909		
EXOSC4	EXOSC4	exosome component 4
LRRFIP1	LRRFIP1	leucine rich repeat (in FLII) interacting protein 1
HIST2H2BB	HIST2H2BB	histone cluster 2, H2bb
SLC26A6	SLC26A6	solute carrier family 26, member 6
KDM2B		
KIF20B		
SPN	SPN	sialophorin (leukosialin, CD43)
TEX22		
TUBB4A		
PUS1	PUS1	pseudouridylate synthase 1
2010012O05RIK		
MSH3	MSH3	mutS homolog 3 (E. coli)
P2RX7	P2RX7	purinergic receptor P2X, ligand-gated ion channel, 7
THAP6	THAP6	THAP domain containing 6
SNRPD3	SNRPD3	small nuclear ribonucleoprotein D3 polypeptide 18kDa
HTATSF1	HTATSF1	HIV-1 Tat specific factor 1
BRWD1	BRWD1	bromodomain and WD repeat domain containing 1
SMC6	SMC6	structural maintenance of chromosomes 6
4921506M07RIK		
L1TD1	L1TD1	LINE-1 type transposase domain containing 1
PLIN4		
TRIO	TRIO	triple functional domain (PTPRF interacting)
TSC1	TSC1	tuberous sclerosis 1
BTAF1	BTAF1	BTAF1 RNA polymerase II, B-TFIID transcription factor-associated, 170kDa (Mot1 homolog, S. cerevisiae)
DDX5	DDX5	DEAD (Asp-Glu-Ala-Asp) box polypeptide 5
SLTM	SLTM	SAFB-like, transcription modulator
PEX6	PEX6	peroxisomal biogenesis factor 6
ZFP259		
2310044G17RIK		
GM19858		
C920008N22RIK		
NAT14	NAT14	N-acetyltransferase 14
9430085M18RIK		
E330018D03RIK		
STK38L	STK38L	serine/threonine kinase 38 like
PLK1S1		
MRPL9	MRPL9	mitochondrial ribosomal protein L9
PAN3	PAN3	PAN3 polyA specific ribonuclease subunit homolog (S. cerevisiae)
CDK11B		
6720401G13RIK		
GM3556		
NFIB	NFIB	nuclear factor I/B
PTPRB	PTPRB	protein tyrosine phosphatase, receptor type, B
ATP2B4	ATP2B4	ATPase, Ca++ transporting, plasma membrane 4
4930558N11RIK		
ULK1	ULK1	unc-51-like kinase 1 (C. elegans)
FBXO30	FBXO30	F-box protein 30
GM14015		
FGD4	FGD4	FYVE, RhoGEF and PH domain containing 4
FAM47E		
GM10142		
ZBTB2	ZBTB2	zinc finger and BTB domain containing 2
STXBP3A		
LOC548102		
D6WSU116E		
C230094B09RIK		
TCF7L1	TCF7L1	transcription factor 7-like 1 (T-cell specific, HMG-box)
A730098P11RIK		
CAMK2B	CAMK2B	calcium/calmodulin-dependent protein kinase (CaM kinase) II beta

Fig 1: heat_map

Heat Map of the top 50 features for each phenotype in LIVER-Normalized FLAG Filtered Data_collapsed_to_symbols

Figure S3D

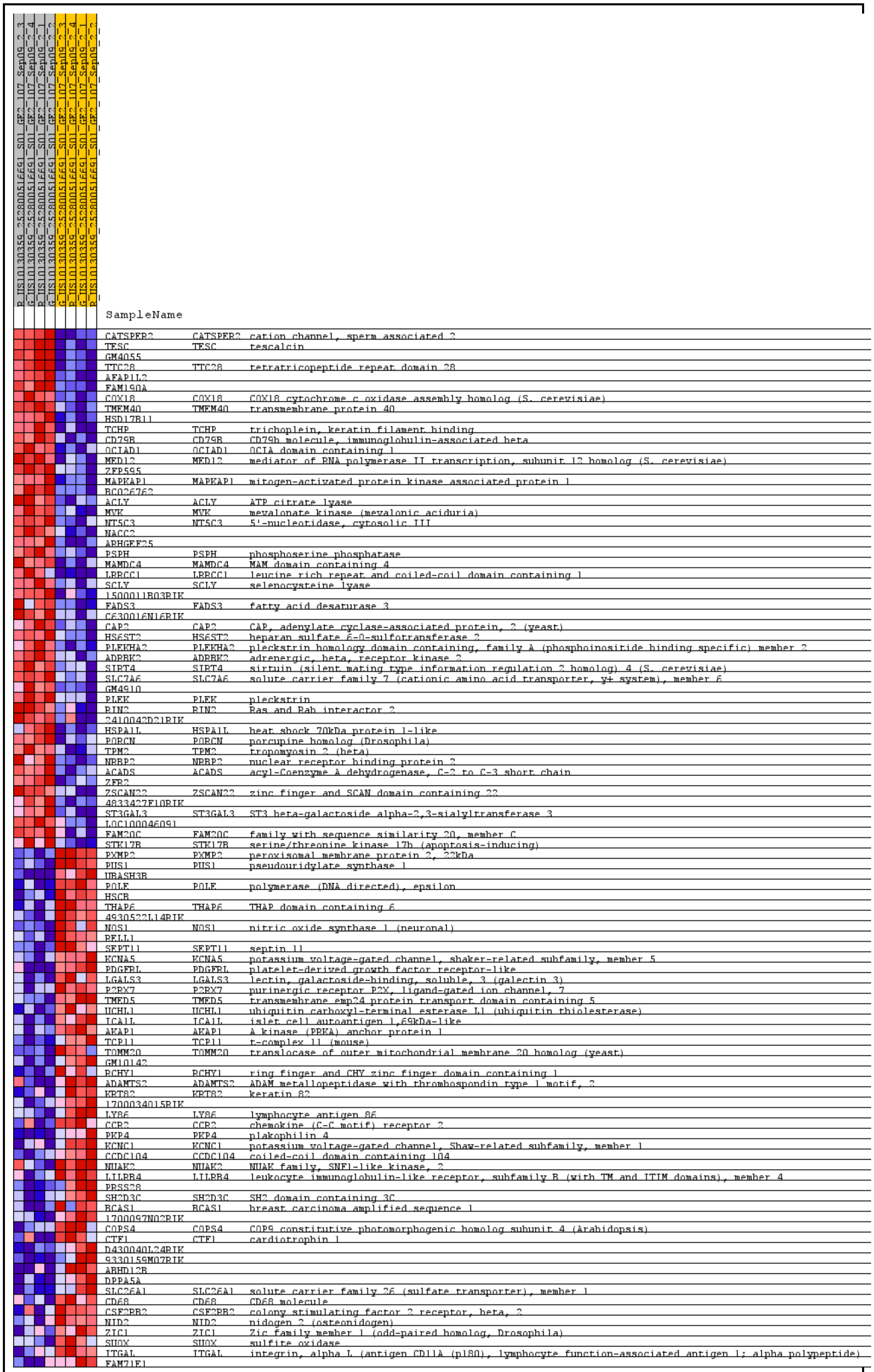

Fig 1: heat_map
Heat Map of the top 50 features for each phenotype in M1_MUSCLE-Normalized FLAG Filtered Data_collapsed_to_symbols

Figure S3E

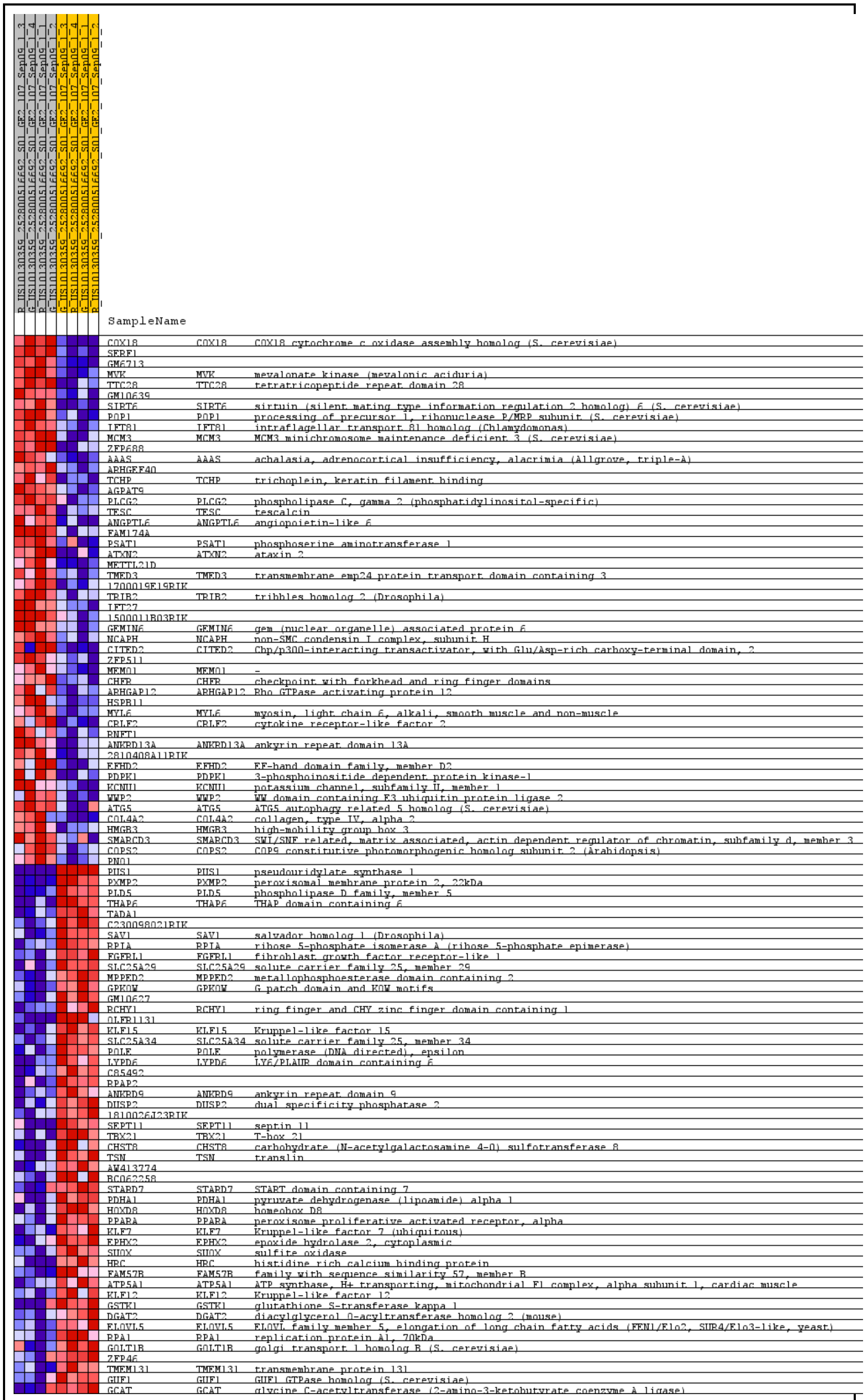

Fig 1: heat_map

Heat Map of the top 50 features for each phenotype in M2_MUSCLE-Normalized FLAG Filtered Data_collapsed_to_symbols

Figure S3F
